# Murine CD4^+^ T Cell Responses Are Inhibited by Cytotoxic T Cell-Mediated Killing of Dendritic Cells and Are Restored by Antigen Transfer

**DOI:** 10.1371/journal.pone.0037481

**Published:** 2012-05-23

**Authors:** Joel Zhi-Iong Ma, So Nai Lim, Jim Shixiang Qin, Jianping Yang, Noriyuki Enomoto, Christiane Ruedl, Franca Ronchese

**Affiliations:** 1 Malaghan Institute of Medical Research, Wellington, New Zealand; 2 School of Biological Sciences, Victoria University of Wellington, Wellington, New Zealand; 3 School of Biological Sciences, Nanyang Technological University, Singapore, Republic of Singapore; University of Bergen, Norway

## Abstract

Cytotoxic T lymphocytes (CTL) provide protection against pathogens and tumors. In addition, experiments in mouse models have shown that CTL can also kill antigen-presenting dendritic cells (DC), reducing their ability to activate primary and secondary CD8^+^ T cell responses. In contrast, the effects of CTL-mediated killing on CD4^+^ T cell responses have not been fully investigated. Here we use adoptive transfer of TCR transgenic T cells and DC immunization to show that specific CTL significantly inhibited CD4^+^ T cell proliferation induced by DC loaded with peptide or low concentrations of protein antigen. In contrast, CTL had little effect on CD4^+^ T cell proliferation induced by DC loaded with high protein concentrations or expressing antigen endogenously, even if these DC were efficiently killed and failed to accumulate in the lymph node (LN). Residual CD4^+^ T cell proliferation was due to the transfer of antigen from carrier DC to host APC, and predominantly involved skin DC populations. Importantly, the proliferating CD4^+^ T cells also developed into IFN-γ producing memory cells, a property normally requiring direct presentation by activated DC. Thus, CTL-mediated DC killing can inhibit CD4^+^ T cell proliferation, with the extent of inhibition being determined by the form and amount of antigen used to load DC. In the presence of high antigen concentrations, antigen transfer to host DC enables the generation of CD4^+^ T cell responses regardless of DC killing, and suggests mechanisms whereby CD4^+^ T cell responses can be amplified.

## Introduction

DC are potent APC that play critical roles in cross-presentation [1] and the differentiation of naïve CD8^+^ T cell into CTL [2]. The development and accumulation of CTL are crucial in controlling and resolving bacterial and viral infections. Pathogen eradication and the pre-determined numerical contraction of specific CTL eventually lead to resolution of the ongoing immune response [3].

The clearance of APC may also contribute to regulating immune responses. Experimental evidence indicates that APC, in particular DC, are targeted and killed by CTL, regulatory T cells, or NK cells [4], [5], [6]. Peptide-specific CTL induced by DC immunization or viral infection *in vivo* [5], [7], or adoptively transferred T cells activated *in vitro* [8] were shown to eliminate antigen-loaded DC *in vivo*. Live imaging of mouse LN confirmed that effector and memory CTL can establish interactions with cognate antigen-loaded DC, and induce DC apoptosis [9]. CTL-mediated killing of DC in turn results in reduced CD8^+^ T cell expansion and anti-tumor immune responses [5], [9], [10], or can modify the phenotype of alloreactive T cell responses [11]. Elimination of DC by regulatory T cells or NK cells was also shown to limit the DC's ability to interact with CD4^+^ and CD8^+^ T cells, and induce their productive activation [4], [12]. Conversely, prolonged DC survival may result in enhanced T cell proliferation, inflammation, and autoimmune manifestations [13], [14].

It has been proposed that the physiological function of CTL-mediated DC killing is to act as a negative feedback mechanism on CD8^+^ T cell immune responses [9], [11], [15]. Antigen presentation to naïve and/or memory CD8^+^ T cells in the LN, and their consequent further expansion, may be unnecessary if effector CTL are already present in the periphery to clear incoming insults. In this scenario the removal of antigen-presenting DC by CTL would be beneficial by preventing redundant T cell expansion [10]. However, it is unclear whether such a regulatory mechanism affects CD4^+^ as well as CD8^+^ T cells, given that they are initiated by different DC subpopulations [1] and are subject to different regulatory mechanisms.

In this article we examine the impact of CTL-mediated DC killing on CD4^+^ T cell responses. We show that proliferation of CD4^+^ T cells was affected by DC killing, and that this was dependent on the amount and form of antigen used to load DC. When antigen presentation remained restricted to the injected DC, CD4^+^ T cell proliferation was inhibited by CTL. In contrast, in conditions where antigenic material was transferred from the injected DC to host DC, CD4^+^ T cell proliferation, memory differentiation and ability to produce IFN-γ were maintained. Therefore, our findings show that inter-DC antigen transfer can influence the size and quality of T cell responses, and suggest antigen transfer as a mechanism to overcome CTL-mediated DC killing.

## Results

### The method of antigen loading affects the sensitivity of DC to CTL-mediated killing, and their ability to accumulate in the draining LN (dLN)

**Figure 1:**
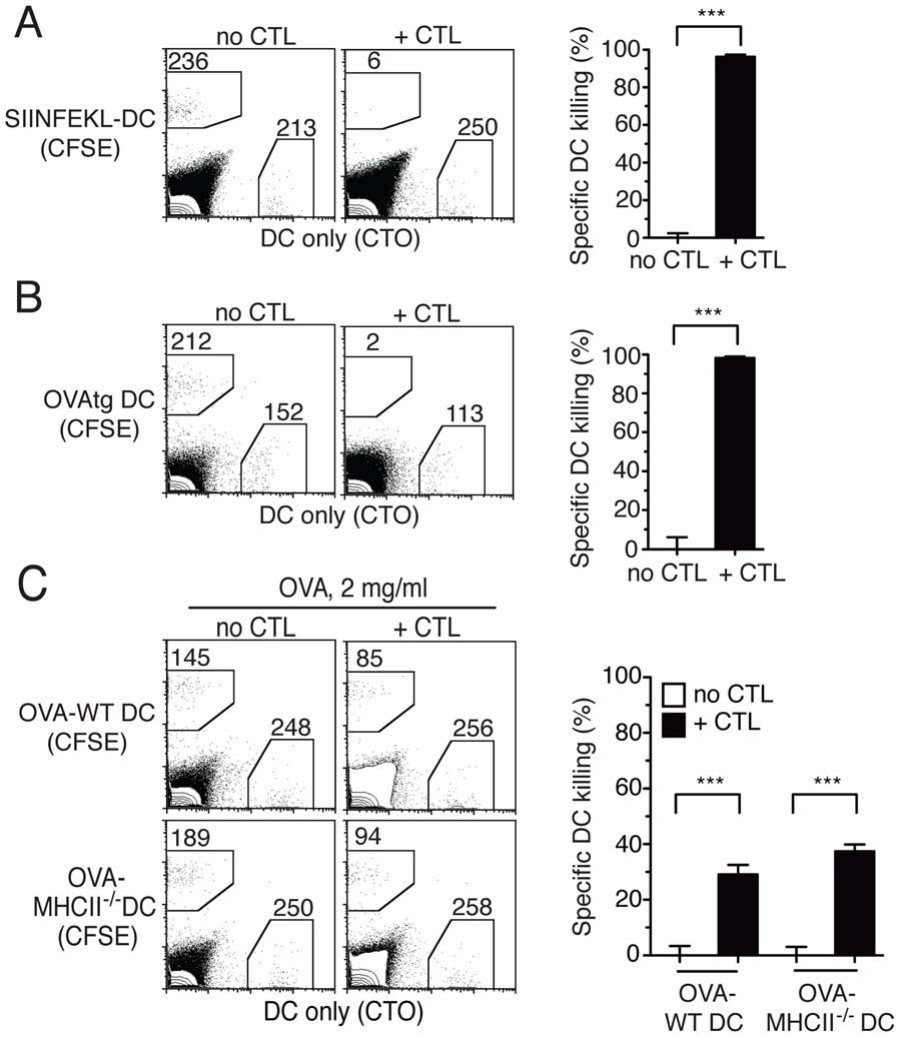
The form of antigen used for loading DC determines sensitivity to CTL-mediated killing in vivo. C57BL/6 mice were injected i.v. with OT-I CTL and challenged s.c. 24 h later with a 1∶1 mix of untreated CTO^+^ DC (DC only), and CFSE^+^ DC loaded with different forms of OVA. Injected DC were recovered from the dLN 48 h later and quantified by flow cytometry. Representative flow cytometry plots from individual dLN are shown on the left; the numbers of events in each gated region are shown. Bar graphs on the right show mean+SEM of OVA-specific DC killing for the indicated groups of mice. (A) Killing of DC loaded with the OVA peptide SIINFEKL (SIINFEKL-DC). The bar graph shows combined results from two experiments each including 2–3 mice per group. (B) Killing of DC endogenously expressing a membrane-associated form of OVA (OVAtg DC). The bar graph shows the data from one of two independent experiments with 3 mice per group that gave similar results. (C) Killing of C57BL/6 or MHCII^−/−^ DC loaded with OVA protein at 2 mg/ml (OVA-DC). The bar graph shows combined results from two experiments each including 2–3 mice per group.

We have reported that effector CTL eliminate DC *in vivo* and prevent their accumulation in the dLN [5]. We used DC from bone marrow (BM) cultures to show that both 5-(and 6)-Carboxyfluorescein diacetate succinimidyl ester (CFSE)-labelled DC loaded with SIINFEKL (SIINFEKL-DC) and Cell Tracker Orange (CTO)-labelled DC not loaded with peptide (DC-only) accumulated in the dLN of naïve mice in equal proportions (Figure 1A). In contrast, the CFSE^+^ SIINFEKL-loaded DC population was selectively depleted in the dLN of mice that had been injected intravenously (i.v.) with *in vitro* activated OT-I CTL 24 h before DC administration. A similar depletion was also observed in C57BL/6 mice that had been immunized to prime an endogenous CTL response [5], [7], [8], [16], indicating that killing of DC can occur in the context of a physiological immune response and does not require CTL transfer.

Peptide incubation is not a physiological method of antigen loading. We therefore tested the sensitivity of DC to CTL-mediated killing using other methods of OVA loading. OVA-transgenic (OVAtg) DC endogenously expressing OVA [17] were eliminated by OT-I CTL as effectively as SIINFEKL-DC (Figure 1B). DC loaded with OVA protein (OVA-DC) at 2 mg/ml, a high dose that is required to obtain cross-presentation by BM DC, were only partially killed by specific CTL (Figure 1C). This reduced killing was not due to the protective effect of CD4^+^ T cells recognizing OVA in the context of MHCII on DC [18], [19], as both C57BL/6 wild type (WT) and MHCII^−/−^ DC were susceptible to CTL killing (Figure 1C). Reduced killing was also not due to some DC not taking up OVA protein, as experiments using fluorochrome-labelled OVA showed that at least 90% of the DC had taken up fluorescent label (not shown). Instead, reduced killing appeared to be due to the relatively inefficient cross-presentation of OVA protein by BM DC, as OVA-DC could induce OT-I proliferation *in vitro* but gave a low to undetectable signal when examined for expression of MHCI/SIINFEKL complexes by staining with the 25-D1 antibody [20] and flow cytometry (not shown). We conclude that the method of antigen loading influences the susceptibility of DC to CTL-mediated killing, presumably by determining the efficiency of MHCI/SIINFEKL complex formation.

### CD4^+^ T cell proliferation in the LN does not require direct presentation by injected DC

By preventing the accumulation of antigen-presenting DC in the dLN, CTL might also inhibit the subsequent induction of CD4^+^ T cell responses. We therefore evaluated the ability of specific CTL to inhibit the division of CFSE-labelled OT-II cells after DC immunization. In all experiments, OT-II T cell division was examined in the dLN 3 days after DC immunization; no division was observed in unimmunized mice, or mice immunized with DC only (not shown).

**Figure 2:**
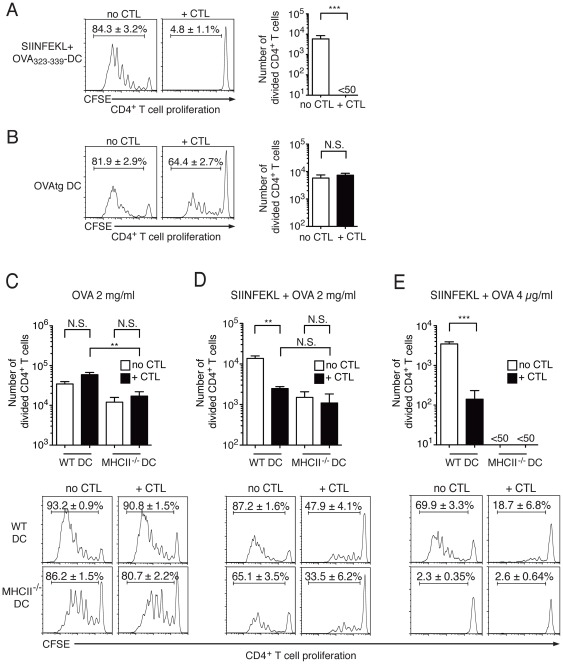
The impact of CTL-mediated killing of DC on CD4^+^ T cell proliferation is determined by the form of antigen loaded on DC. C57BL/6 mice were injected i.v. with CFSE-labelled, CD45-congenic OT-II cells; OT-I CTL were also injected in half of the mice at the same time. 24 h later all mice were immunized s.c. with DC loaded with different forms of OVA. CD4^+^ T cell proliferation in dLN was determined by flow cytometry 3 days after DC immunization. Representative flow cytometry histograms of CD45.1^+^CD4^+^ T cells from individual dLN are on the left of the bar graphs for panels A and B, and below the bar graphs for panels C–E. The mean ± SEM of the percent divided cells for the corresponding group is shown in each panel. Bar graphs show mean+SEM of the number of divided CD45.1^+^CD4^+^ T cells per dLN. (A) CD4^+^ OT-II T cell proliferation in mice immunized with WT DC loaded with SIINFEKL and OVA_323–339_. The bar graph shows data from one of two independent experiments with 5 mice per group that gave similar results. (B) CD4^+^ OT-II T cell proliferation in mice immunized with OVAtg DC. The bar graph shows data from one of two independent experiments with 5 mice per group that gave similar results. (C) CD4^+^ OT-II T cell proliferation in mice immunized with WT DC or MHCII^−/−^ DC loaded with OVA protein at 2 mg/ml. The bar graph shows combined results from two independent experiments each including 5 mice per group. (D) CD4^+^ OT-II T cell proliferation in mice immunized with WT or MHCII^−/−^ DC loaded with SIINFEKL and OVA protein at 2 mg/ml. The bar graph shows results from 5 mice per group. (E) CD4^+^ OT-II T cell proliferation in mice immunized with WT or MHCII^−/−^ DC loaded with SIINFEKL and OVA protein at 4 µg/ml. The bar graph shows combined results from two independent experiments each including 5 mice per group.

Injection of DC loaded with SIINFEKL+OVA_323–339_ induced vigorous OT-II T cell proliferation, but this was reduced to background levels by transfer of OT-I CTL (Figure 2A), indicating that CTL-mediated killing of DC reduced the availability of immunostimulatory antigen in the dLN. Strong OT-II T cell proliferation was also observed in mice immunized with OVAtg DC (Figure 2B), which are highly sensitive to CTL-mediated killing. Again, the percentage of divided OT-II T cells was lower in mice injected with CTL, however, substantial proliferation was still observed and the number of divided OT-II T cells did not significantly differ between untreated and CTL-treated mice (Figure 2B). This result suggests that OVA antigen was still available in the dLN, even when the number of OVAtg DC was decreased to low or undetectable levels due to the presence of specific CTL (Figure 1B).

We then examined OT-II proliferation after immunization with OVA-DC. As shown in Figure 2C, OT-I CTL transfer did not affect the percentage or number of proliferating OT-II cells in dLN. This was not only due to suboptimal killing of OVA-DC by CTL, as simultaneous loading of DC with OVA protein and SIINFEKL led to almost complete killing of DC by CTL (not shown), but did not completely eliminate the proliferation of OT-II cells in the dLN (Figure 2D, compare to Figure 2A).

The results in Figure 2B and 2C suggested that OT-II cell proliferation may not require direct presentation of OVA by injected DC. To assess this possibility, we immunized mice with MHCII^−/−^ DC loaded with OVA protein, as these DC are unable to directly present antigen to OT-II cells. Robust OT-II cell proliferation was observed in mice immunized with OVA-MHCII^−/−^ DC, although the number of divided cells in the dLN was lower than in mice immunized with WT DC (Figure 2C). OT-I CTL transfer did not affect this division. OT-II cell proliferation was also observed in mice immunized with MHCII^−/−^ DC loaded with SIINFEKL+2 mg/ml OVA protein (Figure 2D); interestingly, this proliferation was comparable to the proliferation induced by WT DC in the presence of CTL. Together with the data in Figure 2B, these results suggest that antigenic material can be transferred from the injected DC to host APC, and that host APC can substantially contribute to OT-II cell proliferation.

Other Authors have reported minimal transfer of OVA from injected DC to host DC [21]. To evaluate whether the amount of OVA may underlie this discrepancy, we used a lower protein concentration, 4 µg/ml, which is sufficient for good OT-II cell proliferation *in vitro*. As this OVA concentration is insufficient for cross-presentation by BM DC, DC were also loaded with SIINFEKL to sensitize them to CTL-mediated killing. WT DC loaded with SIINFEKL+OVA protein at 4 µg/ml induced robust OT-II cell division *in vivo*, while MHCII^−/−^ DC loaded with the same amount of OVA induced undetectable responses (Figure 2E), suggesting that presentation by host APC was insufficient for OT-II cell proliferation. Interestingly, in these conditions, OT-I CTL transfer significantly reduced, but did not ablate, OT-II T cell proliferation (Figure 2E).

Taken together, these results suggest that CTL-mediated killing of DC can significantly reduce the magnitude of CD4^+^ T cell responses, if antigen remains localized to the carrier DC (e.g. peptide, low dose protein). In contrast, at high antigen doses, the transfer of antigenic material from the injected DC to other APC enables CD4^+^ T cell proliferation to occur.

### Host DC can take up antigenic material from injected DC in vivo and present it to CD4^+^ and CD8^+^ T cells in vitro

**Figure 3:**
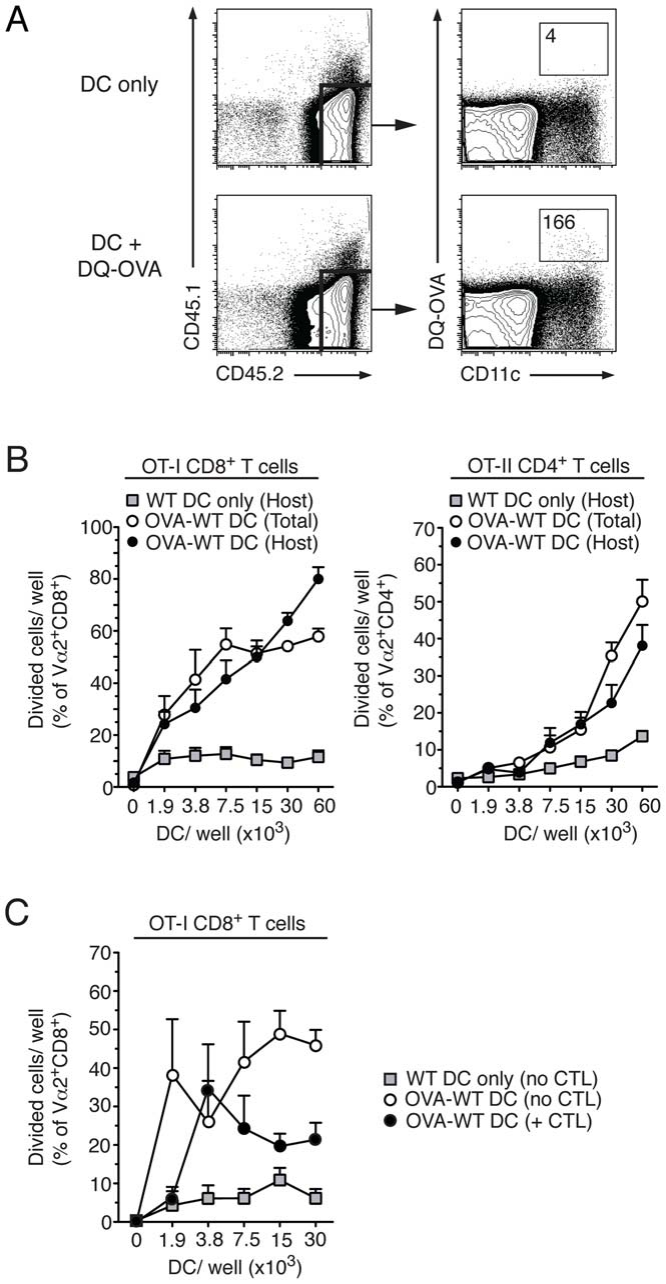
Host DC capture antigen from injected DC and present to CD4^+^ T cells in vitro. A) C57BL/6 mice were immunized with CD45.1^+^ DC loaded with DQ-OVA or no OVA. At 24 h after DC injection, CD45.1^−^CD45.2^+^ host cells in the dLN were identified and examined for CD11c expression and DQ-OVA uptake by flow cytometry. Representative flow cytometry dot plots of live LN cells are shown on the left and CD45.2^+^ cells are shown on the right. The number of events in each gate is shown. (B) C57BL/6 mice were injected with CD45.1^+^ DC, or CD45.1^+^ DC loaded with OVA protein (2 mg/ml). The dLN were collected 24 h later, and the total DC population (donor and host) and CD45.1^−^ DC population (host only) were magnetically purified and cultured in triplicate with CFSE-labelled, purified OT-I or OT-II T for 3 days or 5 days, respectively. OVA-specific proliferation was evaluated as CFSE dilution by flow cytometry. Each symbol shows mean+SEM of the percentage of divided cells/well. Combined data from two independent experiments that gave similar results are shown. (C) As in B, except that some mice were injected i.v. with OT-I CTL 1 day before DC transfer, and only host DC were tested. Symbols shows mean+SEM of the percentage of divided cells/well. Combined data from two independent experiments that gave similar results are shown.

To obtain direct evidence of the transfer of material from injected DC to host APC *in vivo*, CD45.1^+^ DC were incubated with DQ-OVA, which becomes fluorescent only after proteolytic degradation, and injected into CD45.2^+^ mice. As shown in Figure 3A, at 24 h after transfer, DQ-OVA signal could be detected in a CD45.2^+^CD11c^+^ host population in the dLN. This population was not detected in mice injected with unlabelled DC. Therefore, protein carried by the injected DC is taken up by host DC.

Next, we asked if the material captured by host DC could be presented to T cells in an immunostimulatory form. CD45.1^+^ DC loaded with 2 mg/ml OVA protein or no OVA were injected sub-cutaneously (s.c.) into C57BL/6 mice, and 24 h later DC were harvested from dLN and enriched by negative selection. Two DC populations were prepared, a total DC population comprising both host and donor DC, and a host-only DC population where the CD45.1^+^ injected DC were depleted by negative selection. OVA presentation by these two DC populations was determined by co-culturing DC with CFSE-labelled OT-I CD8^+^ or OT-II CD4^+^ T cells for 3 or 5 days, respectively, and examining T cell division by flow cytometry. As shown in Figure 3B, both the total DC and the host-only DC populations induced proliferation of CD8^+^ and CD4^+^ T cells, suggesting that host DC had taken up antigenic material from injected DC and were able to present it in an immunostimulatory form to T cells *in vitro*.

Host DC that take up OVA may cross-present it and become susceptible to CTL-mediated killing *in vivo*. To address this possibility, mice were injected with *in vitro*-activated OT-I CTL before challenge with OVA-DC. DC populations were harvested from dLN 1 day after DC injection and depleted of the injected DC as in the experiment in Figure 3B. As shown in Figure 3C, the ability of host DC to induce proliferation of OT-I T cells *in vitro* was reduced by CTL transfer. This suggests that the number of host DC presenting OVA antigen was also affected by the presence of CTL.

### Antigenic material from injected DC is taken up by host DC in skin and LN

**Figure 4:**
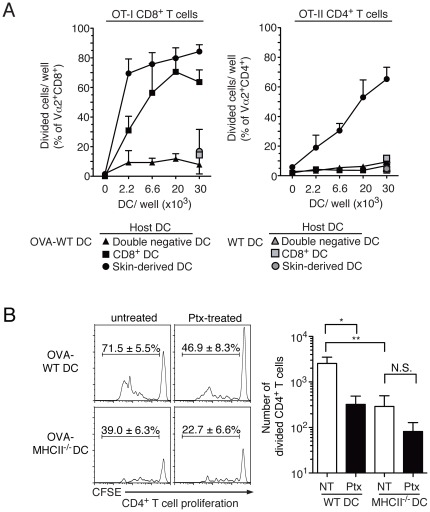
Antigen transfer from injected DC to host DC occurs at the site of DC injection. (A) C57BL/6 mice were injected with CD45.1^+^ WT DC or CD45.1^+^ WT DC loaded with OVA protein (2 mg/ml). The dLN were collected 24 h later, and the CD8^+^ DC, the CD8^−^CD205^+^ skin-derived DC and the CD8^−^CD205^−^ double negative DC populations were sorted and cultured in duplicate with CFSE-labelled, purified OT-I or OT-II T for 3 days or 5 days, respectively. OVA-specific proliferation was evaluated as CFSE dilution by flow cytometry. Each symbol represents the mean+SEM of the percentage of divided cells/well. Combined data from two independent experiments that gave similar results are shown. (B) C57BL/6 were injected with CFSE-labelled CD45-congenic OT-II CD4^+^ T cells and immunized 24 h later with WT or MHCII^−/−^ DC that had been loaded with OVA protein (2 mg/ml), and treated with Ptx or left untreated. CD4^+^ T cell proliferation in dLN was determined by flow cytometry 3 days after DC immunization. Representative flow cytometry histograms of CD45.1^+^CD4^+^ T cells from individual dLN are shown on the left. The mean ± SEM of the percent divided cells in each group is shown. The bar graph on the right shows mean+SEM of the number of divided CD45.1^+^CD4^+^ T cells/dLN. Combined results from two independent experiments each with 5 mice per group are shown.

To identify the host DC subset that was presenting OVA from the injected DC to T cells *in vitro*, host DC were purified by flow sorting into a CD205^+^CD8^lo^ skin-derived population, a CD205^+^CD8^hi^ LN-resident population, and a CD205^−^CD8^−^ (double negative) DC population, and each population was tested for presentation of OVA to OT-I and OT-II cells *in vitro*. Host skin-derived DC were dominant in presenting OVA to OT-I CD8^+^ T cells, and were the only subset tested that induced proliferation of OT-II CD4^+^ T cells (Figure 4A). LN-resident CD8^+^ DC induced proliferation of OT-I CD8^+^ T cells, but not OT-II CD4^+^ T cells, while the double-negative DC populations did not induce proliferation of either T cell population. We conclude that OVA carried by injected DC is transferred to host skin-derived DC, and can be presented by these cells to induce CD4^+^ T cell proliferation *in vitro*.

We wished to determine the site of OVA transfer from injected DC to host skin DC, and used pertussis toxin (Ptx) to block DC migration from the injection site to the dLN [22]. Preliminary experiments showed that Ptx treatment did not affect DC viability, and was effective at inhibiting DC migration to the dLN (data not shown). Ptx-treated DC were loaded with a high dose of OVA, 2 mg/ml, and injected s.c. As shown in Figure 4B, OT-II T cell proliferation after immunization with Ptx-treated DC was lower than in mice immunized with untreated DC, but clearly detectable and comparable in magnitude to the proliferation induced by MHCII^−/−^ DC loaded with the same amount of OVA. The induction of OT-II T cell proliferation by MHCII^−/−^ DC was further decreased by Ptx treatment, but this decrease was not statistically significant (Figure 4B). As Ptx-treated DC are unable to reach the dLN, these results suggest that migration to the dLN is not necessary for the transfer of antigenic material from injected DC to host DC.

### The differentiation of memory CD4^+^ T cells is not prevented by CTL-mediated DC killing

**Figure 5:**
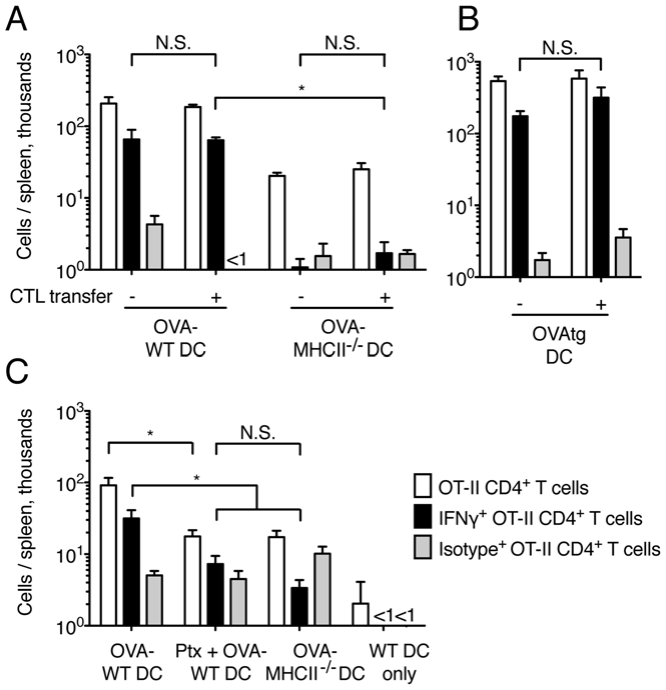
Induction of IFN-γ production by CD4^+^ T is not prevented by CTL-mediated DC killing. C57BL/6 mice were injected with CD45-congenic OT-II CD4^+^ T cells; OT-I CTL were injected in some of the mice at the same time. 24 h later, mice were immunized s.c. with DC loaded with different forms of OVA. 19 days after immunization, spleens were collected and the total number of OVA-specific, IFN-γ-producing CD4^+^ T cells was determined by intracellular staining. Bar graphs show mean+SEM. (A) Mice were immunized with WT or MHCII^−/−^ DC loaded with OVA protein (2 mg/ml). The bar graph shows data from one of two similar experiments, each with 5 mice per group, that gave similar results. (B) Mice were immunized with OVAtg DC. Results are from 5 mice per group. (C) Mice were immunized with WT or MHCII^−/−^ DC loaded with OVA protein (2 mg/ml); some of the DC were also treated with Ptx. The bar graph shows data from 5 mice per group.

OT-I CTL did not inhibit the proliferation of OT-II T cells after immunization with OVA-DC (Figure 2C). We wished to determine whether this initial proliferation also resulted in the generation of memory cells and ability to produce cytokines, and therefore assessed IFN-γ production by spleen CD4^+^ cells 19 days after DC immunization [23]. In mice immunized with WT DC loaded with 2 mg/ml OVA, OT-II T cells were clearly detectable in the spleen and approximately one quarter of these also produced IFN-γ upon *in vitro* restimulation. Neither the number of OT-II cells nor their ability to produce IFN-γ were affected by transfer of OT-I CTL (Figure 5A). In contrast, as also reported by other Authors [21], immunization with MHCII^−/−^ DC induced lower numbers of spleen OT-II cells compared to immunization with WT OVA-DC, and only about 10% of these OT-II cells produced IFN-γ. Transfer of OT-I CTL did not affect either of these responses (Figure 5A).

To test the effects of CTL on CD4^+^ T cell responses under more stringent conditions, we also used OVAtg DC as their numbers in dLN were substantially reduced by CTL transfer (Figure 1B). Again, CTL transfer did not affect the frequency of OT-II cells in the spleen, nor their ability to produce IFN-γ when restimulated *in vitro* (Figure 5B). Thus, CTL-mediated DC killing does not impair the induction of IFN-γ-producing OT-II T cells after DC immunization.

Pre-treating OVA-DC with Ptx to inhibit their migration to the dLN also caused a reduction of both the number of OT-II cells in the spleen, and their ability to produce IFN-γ (Figure 5C). This response was not significantly different from the response induced by MHCII^−/−^ DC. Thus, antigen presentation outside the dLN was also not sufficient for the induction of IFN-γ-producing OT-II cells (Figure 5C).

## Discussion

In this study we investigate the effects of CTL-mediated killing of antigen-presenting DC on the development of CD4^+^ T cell responses. We used adoptive transfer of TCR transgenic T cells and DC loaded with different forms of antigen to establish that antigen-presenting DC injected *in vivo* were variably sensitive to CTL-mediated killing, and that sensitivity to killing correlated with the expected efficiency of MHCI/SIINFEKL complex formation. Unexpectedly, we also observed that the ability of CTL to kill DC *in vivo* was not sufficient to predict inhibition of CD4^+^ T cell responses. In conditions of high antigen loading, which permit transfer of antigenic material from injected DC to host DC, OT-II T cell proliferation and memory differentiation were preserved, even if the injected DC appeared to be effectively cleared by CTL and were not detected in the dLN.

Previous studies have examined the effects of CTL-mediated killing of DC on CD4^+^ T cell responses. Guarda *et al.* have reported that CTL can suppress the proliferation of naïve CD4^+^ T cells after immunization with peptide-loaded DC [9], while Laffont *et al* reported changes in the Th1/Th2 phenotype of allogeneic T cells induced by DC immunization [11]. We wished to extend those studies to DC loaded with antigen in different forms and amounts, to establish the conditions in which DC are affected by CTL-mediated killing. We observed that CD4^+^ T cell responses induced by immunization with peptide-loaded DC were the most sensitive to the inhibitory effect of CTL, a results presumably due to the combination of the high expression of MHCI/SIINFEKL complexes at the cell surface, and consequent efficient recognition by CTL, with a relatively inefficient transfer of antigenic material to host APC. At the opposite end of the spectrum, CD4^+^ T cell responses induced by DC loaded with very high amounts of OVA protein were only weakly affected by CTL. This finding was only partly due to inefficient cross-presentation of OVA on MHCI, as loading these DC with SIINFEKL peptide to make them more sensitive to CTL-mediated killing was not sufficient to fully restore the sensitivity of the CD4^+^ response to CTL-mediated inhibition, but reduced it to a level similar to that observed after immunization with MHCII^−/−^ DC (Figure 2D). Thus, transfer of antigenic material from the carrier DC to host APC appeared to play a key role in the generation of these remaining CD4^+^ T cell responses.

Transfer of antigenic material to host APC required high antigen doses, which are unlikely to be available *in vivo* under physiological situations. In contrast, lower OVA concentrations (4–20 µg/ml) were insufficient for transfer, and could induce CD4^+^ T cell proliferation only if directly presented by carrier DC. While these low OVA concentrations were not cross-presented by the BM DC used in our study, they approach a range that can be cross-presented by specialized DC populations, or if modified to facilitate uptake [24], suggesting that DC loaded with physiological amounts of antigen can be recognized and killed by specific CTL. This possibility is supported by our recent data showing that OVA-specific CTL can suppress Th2 effector responses in the airway [16], presumably through killing of antigen-presenting DC.

Antigen transfer between injected DC and host DC has been reported previously [21], [25], [26], [27], [28]. In line with those reports, we show here that DQ-OVA, but also FITC-dextran or intracellular labelling with CFSE or CTO (data not shown), carried by injected DC could be detected in a small proportion of host DC, providing evidence for the transfer of antigenic material *in vivo*. Transwell experiments using OVAtg DC (not shown) also indicated that antigen transfer did not require direct contact between “carrier” and “host” DC, suggesting that it may be mediated via transfer of cellular fragments or exosomes. Transfer of MHC-peptide complexes has also been documented in other studies [29]. While this DC “cross-dressing” would not be revealed in the assays described above, it may also contribute to our findings.

Host DC prepared from the dLN of mice injected with OVA-DC could cross-present OVA to OT-I T cells *in vitro*. Similar results were obtained also using SIINFEKL-DC (not shown), suggesting that antigen and/or antigen-MHC complexes could both transfer from injected DC to host DC. In line with those observations, results in Figure 3C show that the stimulatory ability of host DC was decreased by CTL transfer *in vivo*, presumably via the killing of antigen presenting DC. Such killing is consistent with our previous reports that antigen-specific CTL can inhibit the expansion of naïve [5] and antigen-experienced [10] CD8^+^ T cells *in vivo* by killing antigen-presenting DC. However, CTL failed to completely block CD4^+^ T cell proliferation induced by DC loaded with OVA protein (Figure 2B–E). These results suggest that, on a per cell basis, some DC may escape CTL-mediated killing by failing to simultaneously present antigen in the context of both MHCI and MHCII.

Host skin-derived DC could present OVA to both CD4^+^ and CD8^+^ T cells *in vitro*, whereas host LN-resident CD8^+^ DC only induced proliferation of OVA-specific CD8^+^ T cells. The observation that skin-derived host DC could present antigen from injected DC suggests that antigen transfer was already occurring at the site of DC injection in the skin. This conclusion is also supported by experiments showing that host DC could stimulate CD4^+^ T cell proliferation *in vivo*, even when migration of the injected DC to the dLN was inhibited using Ptx. These results differ from previous studies where injected DC transferred their antigens to host DC in the dLN [25], and suggest the existence of multiple mechanisms of antigen exchange between DC populations. In contrast to MHCII, some MHCI-restricted antigen transfer between migratory DC and CD8^+^ DC also occurred in the dLN. Transfer of antigen from tissue-derived migratory DC to resident DC within the LN has been documented in a viral infection model [30]. In our experiments, both skin-derived DC and injected DC could potentially transport antigens to CD8^+^ DC because their migratory route through the lymphatics takes them into the outer paracortex where CD8^+^ DC reside [31], [32]. We have yet to determine which population is the source of this antigen.

Immunization with antigen-loaded DC not expressing the appropriate MHCII molecules has been shown to generate weak CD4^+^ T cell responses [21], [25]. We also found that immunization with MHCII^−/−^ DC, or Ptx-treated DC, was sufficient for early CD4^+^ T cell proliferation in dLN, but did not induce memory responses or IFN-γ-producing OT-II T cells in spleen, possibly suggesting that, after early division, OT-II T cells failed to fully expand or underwent deletional tolerance. In contrast, CTL transfer appeared to strongly reduce the accumulation of DC – and hence direct antigen presentation – in dLN, but did not affect CD4^+^ memory formation or the generation of IFN-γ-producing OT-II T cells. The mechanism of this finding is not yet established. CTL may not completely prevent the accumulation of injected DC in the dLN, and DC numbers that are too low or too transient to be detected in our experiments might still be sufficient for full CD4^+^ T cell responses. We think this is an unlikely possibility: other Authors have reported that continued antigen presentation is required for optimal CD4^+^ T cell responses *in vivo* [33], [34], [35]. Therefore, by itself, this scenario appears unlikely to fully explain our results. A perhaps more likely possibility is that host DC may co-operate with the few injected DC that reach the dLN to induce full CD4^+^ T cell responses. CTL-derived cytokines induced by recognition of antigen on injected DC [36] may support this process, perhaps by promoting host DC maturation and migration to the dLN [37], [38]. This possibility is consistent with the observation that MHCII^−/−^ DC are unable to induce IFN-γ responses regardless of the presence of CTL. While cytokine exposure is not thought to be sufficient to enable DC to induce effector differentiation of CD4^+^ T cells [39], it may provide sufficient signals for DC to support memory development and some IFN-γ production, as observed in our experiments.

The importance of CTL and NK cell-mediated killing in the regulation of DC survival and the control of immune responses is consistent with observations that perforin deficiencies, which dramatically affect cytotoxic function, are associated with immune dysregulation and increased immune responses in mice and humans [40], [41]. Together with our recent work [16], data in this paper suggest that DC killing can also affect, although variably, CD4^+^ T cell responses *in vivo*, possibly explaining why perforin mutations are associated with accumulation of activated CD8^+^ and also CD4^+^ T cells [42], [43].

A better understanding of the conditions that control inhibition vs. maintenance of T cell responses in the face of pre-existing CTL and clearance of antigen-presenting DC will be important in the understanding of how immune responses are maintained or resolved, and in the design of protocols of DC administration for the purpose of immunotherapy. Inter-DC antigen transfer has been reported to result in tolerance induction in CD4^+^ T cells in the steady state [44], while in the context of a viral infection it may lead to amplified CD8^+^ immune responses [30]. Others have reported that ‘cross-dressed’ DC, presenting pre-formed antigen-MHCI complexes acquired from other cells, can activate memory CD8^+^ T cells [29], supporting the notion that inter-DC antigen transfer can provide antigenic stimulation for memory T cells. We look forward to studies where the physiological importance of CTL-mediated DC killing and inter-DC antigen transfer is thoroughly evaluated, and exploited for the purpose of improved immunotherapies.

## Materials and Methods

### Ethics statement

All experimental protocols were approved by the Victoria University Animal Ethics Committee (permits No. 2007R3M and 2010R2M) and performed in accordance with Institutional guidelines.

### Mice

All mice were bred and maintained at the Malaghan Institute of Medical Research Biomedical Research Unit. C57BL/6 and CD45-congenic B6.SJL-*Ptprc* ^a^ were from the Animal Resources Centre, Perth, Australia, and MHCII^−/−^ B6Aa^0^/Aa^0^ [45] from Dr. Horst Blüthmann, Hoffmann-La Roche, Basel, Switzerland. OT-I [46] and OT-II [47] mice carrying transgenic TCR specific for K^b^+OVA_257–264_ and I-A^b^+OVA_323–339_, respectively, were from Prof. F. Carbone, University of Melbourne, Melbourne, Australia.

### In vitro culture media and reagents

All cultures were in complete Iscove's Modified Dulbecco Medium (cIMDM) consisting of IMDM supplemented with 5% FCS, 100 U/ml penicillin, 100 µg/ml streptomycin and 55 µM 2-ME (all from Invitrogen, USA). Recombinant cytokines were prepared from transfected cell lines and titrated using commercial standards as a reference.

### Cell lines and DC cultures

DC were generated from C57BL/6 BM or Nup98 HoxB4 OVAtg hematopoietic stem cells by culturing for 7 days in 10 ng/ml murine rGM-CSF and 20 ng/ml murine rIL-4 as described [48]. The Nup98 HoxB4 OVAtg haematopoietic stem cell line was prepared from OVAtg mice [17] and maintained in culture as described [49].

### DC loading with antigen and injection in vivo

Non-adherent cells were harvested from GM-CSF/IL-4 cultures on day 5 and incubated in fresh plates with the indicated concentration of Grade V OVA (Sigma-Aldrich) for a further 48 h. In some experiments, endotoxin-free OVA (Profos AG, Regensburg, Germany) or DQ-OVA (Molecular Probes, Invitrogen) were used. Lipopolysaccharide (LPS, Sigma-Aldrich) was added at 100 ng/ml during the last 18–24 h of culture, after which DC were washed and injected at 2×10^5^ cells/mouse. OVAtg DC were activated with LPS and injected using these same conditions.

For peptide loading, LPS-treated DC were loaded with 1–10 µM OVA_257–264_ (SIINFEKL) and/or OVA_323–339_ (ISQAVHAAHAEINEAGR) peptides (both from Mimotope, Australia) for 4 h at 37°C, and washed twice before injection. In some experiments, DC were simultaneously treated with pertussis toxin (List Biological Laboratories, Inc, USA) at 20 ng/ml for 2–3 h. Peptide-loaded DC were resuspended in IMDM and injected at 1×10^5^ cells/mouse.

All DC were injected s.c. in the volar aspect of the anterior forelimb. using a 29 G needle and a 0.3 ml syringe (BD Biosciences, USA).

### In vitro OT-I T cell activation and transfer

OT-I T cells were activated *in vitro* by culturing with DC and 0.1 µM SIINFEKL for 4 days, and expanded in 100 U/ml IL-2 for a further 2 days as described [50]. Greater than 95% of the resulting T cells were Vα2^+^Vβ5.1/5.2^+^ and CD8^+^CD62L^lo^CD44^hi^. 5×10^6^ OT-I CTL were transferred i.v. into each mouse.

### In vivo DC migration and cytotoxicity assays

LPS-treated GM-CSF/IL-4 DC were labelled with 1 µM CFSE (Molecular Probes, USA) or 10 µM CTO (Molecular Probes) as described [7], mixed, and 0.5×10^6^ of each population were injected s.c. into the forelimb. At different times after injection, brachial and axillary LN were harvested and digested with 100 µg/ml DNase I (Roche, USA) and 0.1 mg/ml Liberase CI (Roche, USA) for 25 min and a subsequent 5 min with 10 mM EDTA at 37°C. DC numbers in dLN were determined by flow cytometry [7], [10].

### Enrichment and labelling of CD4^+^ and CD8^+^ T cells

Naïve OT-II and OT-I T cells were prepared from LN and spleen cell suspensions and positively selected using anti-CD4 and anti-CD8 magnetic beads (Miltenyi Biotech GmBH, Germany), respectively. Enriched T cells were labelled with 1 µM CFSE for 10 mins at 37°C; labelling was stopped by adding FCS and IMDM and cells were washed extensively before injection.

### In vivo T cell proliferation

CFSE-labelled OT-II CD4^+^ T cells were injected i.v. 24 h prior to DC immunization. DC not loaded with OVA were included as negative control in each experiment. Three days after DC injection, OT-II CD4^+^ T cell proliferation was assessed in the pooled ipsilateral axillary and brachial LN using flow cytometry.

### Ex vivo DC isolation and sorting

*Ex vivo* DC isolation and sorting were adapted from [51], [52]. Brachial and axillary LN were digested with 100 µg/ml DNase I and 0.3 mg/ml Liberase CI for 25 min at 37°C, followed by EDTA (10 mM), and incubated with anti-FcγRII/III (2.4G2) for 15–20 min at 4°C. Cell suspensions were then incubated with biotinylated anti-CD3ε (145-2C11), anti-NK1.1 (PK136), anti-DX5 (DX5), anti-Ter119 (Ter119), anti-Thy1.2 (53-2.1) (all from eBiosciences, USA), anti-CD19 (1D3), anti-Gr-1 (RB6-8C5) (BD Pharmingen, USA) and anti-B220 (6B2; purified in-house) for 30 min at 4°C. In some experiments, biotinylated anti-CD45.1 (A20; eBiosciences) was also added. Antibody-labelled cells were depleted using streptavidin magnetic beads and an AutoMACS (both from Miltenyi Biotech GmBH, Germany) to obtain populations that were 60–80% CD11c^+^. To separate DC subsets, CD11c^+^ cells were first sorted on high CD11c expression (HL3; BD Pharmingen), followed by sorting for CD205 (205yekta; eBiosciences) and CD8 (53-6.7; BD Pharmingen) using a FACSVantage SE (BD Biosciences). The purity of sorted DC subsets was over 94%. Sorted DC were then cultured with CFSE-labelled OT-I CD8^+^ T cells or OT-II CD4^+^ T cells for 3 or 5 days, respectively, to evaluate antigen presentation.

### In vitro T cell restimulation assays and intracellular staining for cytokines

Spleens from immunized mice were digested with 100 µg/ml DNase I and 0.1 mg/ml Liberase CI for 25 min, followed by 10 mM EDTA at 37°C; RBC were then lysed. Spleen cell suspensions were plated in 6-well plates at 6×10^6^ cells/well in cIMDM with or without 10 µM OVA_323–339_ and incubated for 15 h. After an additional 5 h incubation with GolgiStop (BD Pharmingen) at 37°C, cells were surface labelled, permeabilized with BD Cytofix/Cytoperm (BD Pharmingen) and labelled with anti-IFN-γ (XMG1.2; BD Pharmingen) or isotype control (R3-34; BD Pharmingen). Cells were washed thrice with BD Perm/Wash buffer before flow cytometry.

### Flow cytometry analysis

Anti-FcγRII/III receptor (2.4G2), anti-CD4 (GK1.5), anti-CD8α (2.43) and anti-CD11c (N418) antibodies were affinity-purified from hybridoma culture supernatants and conjugated to allophycocyanin (APCy) as indicated. Anti-Vα2-PE (B20.1), anti-CD45.1-PE (A20), anti-CD4-FITC (GK1.5), anti-CD8α-APCy-H7 (53-6.7), anti-IFN-γ-APCy (XMG1.2) and Rat IgG1 κ isotype-APCy (R3-34) were from BD Pharmingen (USA). Anti-CD11c-PE-Cy7 (N418), anti-CD45.1-PerCP-Cy5.5 (A20) and anti-CD45.2 (104) conjugated to PE-Cy5.5, PE and APCy were from eBiosciences (USA). Anti-CD45.2-Pacific blue (104) and anti-CD11c-Alexa-Fluor 700 (N418) were from Biolegend (USA). All samples were analysed on a FACSort, FACScalibur, or LSRII SORP flow cytometer (BD Biosciences, USA) using CellQuest. Dead cells were excluded from analysis using propidium iodide (BD Pharmingen, USA) or DAPI (Molecular Probes, USA) labelling. FlowJo version 9.0.2 (Treestar Inc, USA) was used for analysis.

### Statistical analysis

Statistical analysis was conducted using GraphPad Prism software using the Student's *t*-test for comparison between two groups, or one-way ANOVA with Bonferroni's correction for multiple group comparisons. Experimental data sets were tested using the D'Agostino and Pearson omnibus test and shown to be consistent with a Gaussian distribution. Means+SEM are shown in all graphs; for the sake of clarity, one-sided error bars are shown.

## References

[1] Heath WR, Carbone FR (2001). Cross-presentation, dendritic cells, tolerance and immunity.. Annu Rev Immunol.

[2] Jung S, Unutmaz D, Wong P, Sano G, De los Santos K (2002). In vivo depletion of CD11c+ dendritic cells abrogates priming of CD8+ T cells by exogenous cell-associated antigens.. Immunity.

[3] Williams MA, Bevan MJ (2007). Effector and memory CTL differentiation.. Annu Rev Immunol.

[4] Boissonnas A, Scholer-Dahirel A, Simon-Blancal V, Pace L, Valet F (2010). Foxp3+ T cells induce perforin-dependent dendritic cell death in tumor-draining lymph nodes.. Immunity.

[5] Hermans IF, Ritchie DS, Yang J, Roberts JM, Ronchese F (2000). CD8+ T cell-dependent elimination of dendritic cells in vivo limits the induction of antitumor immunity.. J Immunol.

[6] Laffont S, Seillet C, Ortaldo J, Coudert JD, Guery JC (2008). Natural killer cells recruited into lymph nodes inhibit alloreactive T-cell activation through perforin-mediated killing of donor allogeneic dendritic cells.. Blood.

[7] Ritchie DS, Hermans IF, Lumsden JM, Scanga CB, Roberts JM (2000). Dendritic cell elimination as an assay of cytotoxic T lymphocyte activity in vivo.. J Immunol Methods.

[8] Andrew KA, Simkins HM, Witzel S, Perret R, Hudson J (2008). Dendritic cells treated with lipopolysaccharide up-regulate serine protease inhibitor 6 and remain sensitive to killing by cytotoxic T lymphocytes in vivo.. J Immunol.

[9] Guarda G, Hons M, Soriano SF, Huang AY, Polley R (2007). L-selectin-negative CCR7- effector and memory CD8+ T cells enter reactive lymph nodes and kill dendritic cells.. Nat Immunol.

[10] Yang J, Huck SP, McHugh RS, Hermans IF, Ronchese F (2006). Perforin-dependent elimination of dendritic cells regulates the expansion of antigen-specific CD8+ T cells in vivo.. Proc Natl Acad Sci U S A.

[11] Laffont S, Coudert JD, Garidou L, Delpy L, Wiedemann A (2006). CD8+ T-cell-mediated killing of donor dendritic cells prevents alloreactive T helper type-2 responses in vivo.. Blood.

[12] Andrews DM, Estcourt MJ, Andoniou CE, Wikstrom ME, Khong A (2010). Innate immunity defines the capacity of antiviral T cells to limit persistent infection.. J Exp Med.

[13] Chen M, Felix K, Wang J (2012). Critical role for perforin and Fas-dependent killing of dendritic cells in the control of inflammation.. Blood.

[14] Chen M, Wang YH, Wang Y, Huang L, Sandoval H (2006). Dendritic cell apoptosis in the maintenance of immune tolerance.. Science.

[15] Ronchese F, Hermans IF (2001). Killing of dendritic cells: a life cut short or a purposeful death?. J Exp Med.

[16] Enomoto N, Hyde E, Ma JZ, Yang J, Forbes-Blom E (2012). Allergen-specific CTL require perforin expression to suppress allergic airway inflammation.. J Immunol.

[17] Ehst BD, Ingulli E, Jenkins MK (2003). Development of a novel transgenic mouse for the study of interactions between CD4 and CD8 T cells during graft rejection.. Am J Transplant.

[18] Medema JP, Schuurhuis DH, Rea D, van Tongeren J, de Jong J (2001). Expression of the serpin serine protease inhibitor 6 protects dendritic cells from cytotoxic T lymphocyte-induced apoptosis: differential modulation by T helper type 1 and type 2 cells.. J Exp Med.

[19] Mueller SN, Jones CM, Stock AT, Suter M, Heath WR (2006). CD4+ T cells can protect APC from CTL-mediated elimination.. J Immunol.

[20] Porgador A, Yewdell JW, Deng Y, Bennink JR, Germain RN (1997). Localization, quantitation, and in situ detection of specific peptide-MHC class I complexes using a monoclonal antibody.. Immunity.

[21] Kuipers H, Soullie T, Hammad H, Willart M, Kool M (2009). Sensitization by intratracheally injected dendritic cells is independent of antigen presentation by host antigen-presenting cells.. J Leukoc Biol.

[22] Itano AA, McSorley SJ, Reinhardt RL, Ehst BD, Ingulli E (2003). Distinct dendritic cell populations sequentially present antigen to CD4 T cells and stimulate different aspects of cell-mediated immunity.. Immunity.

[23] Reinhardt RL, Khoruts A, Merica R, Zell T, Jenkins MK (2001). Visualizing the generation of memory CD4 T cells in the whole body.. Nature.

[24] Dickgreber N, Stoitzner P, Bai Y, Price KM, Farrand KJ (2009). Targeting antigen to MHC class II molecules promotes efficient cross-presentation and enhances immunotherapy.. J Immunol.

[25] Kleindienst P, Brocker T (2003). Endogenous dendritic cells are required for amplification of T cell responses induced by dendritic cell vaccines in vivo.. J Immunol.

[26] Luketic L, Delanghe J, Sobol PT, Yang P, Frotten E (2007). Antigen presentation by exosomes released from peptide-pulsed dendritic cells is not suppressed by the presence of active CTL.. J Immunol.

[27] Petersen TR, Sika-Paotonu D, Knight DA, Simkins HM, Hermans IF (2011). Exploiting the Role of Endogenous Lymphoid-Resident Dendritic Cells in the Priming of NKT Cells and CD8+ T Cells to Dendritic Cell-Based Vaccines.. PLoS One.

[28] Yewdall AW, Drutman SB, Jinwala F, Bahjat KS, Bhardwaj N (2010). CD8+ T cell priming by dendritic cell vaccines requires antigen transfer to endogenous antigen presenting cells.. PLoS One.

[29] Wakim LM, Bevan MJ (2011). Cross-dressed dendritic cells drive memory CD8+ T-cell activation after viral infection.. Nature.

[30] Allan RS, Waithman J, Bedoui S, Jones CM, Villadangos JA (2006). Migratory dendritic cells transfer antigen to a lymph node-resident dendritic cell population for efficient CTL priming.. Immunity.

[31] Kissenpfennig A, Henri S, Dubois B, Laplace-Builhe C, Perrin P (2005). Dynamics and function of Langerhans cells in vivo: dermal dendritic cells colonize lymph node areas distinct from slower migrating Langerhans cells.. Immunity.

[32] Bajenoff M, Granjeaud S, Guerder S (2003). The strategy of T cell antigen-presenting cell encounter in antigen-draining lymph nodes revealed by imaging of initial T cell activation.. J Exp Med.

[33] Blair DA, Turner DL, Bose TO, Pham QM, Bouchard KR (2011). Duration of antigen availability influences the expansion and memory differentiation of T cells.. J Immunol.

[34] Jusforgues-Saklani H, Uhl M, Blachere N, Lemaitre F, Lantz O (2008). Antigen persistence is required for dendritic cell licensing and CD8+ T cell cross-priming.. J Immunol.

[35] Obst R, van Santen HM, Mathis D, Benoist C (2005). Antigen persistence is required throughout the expansion phase of a CD4(+) T cell response.. J Exp Med.

[36] Hufford MM, Kim TS, Sun J, Braciale TJ (2011). Antiviral CD8+ T cell effector activities in situ are regulated by target cell type.. J Exp Med.

[37] Brehm MA, Daniels KA, Welsh RM (2005). Rapid production of TNF-alpha following TCR engagement of naive CD8 T cells.. J Immunol.

[38] Min L, Mohammad Isa SA, Shuai W, Piang CB, Nih FW (2010). Cutting edge: granulocyte-macrophage colony-stimulating factor is the major CD8+ T cell-derived licensing factor for dendritic cell activation.. J Immunol.

[39] Sporri R, Reis e Sousa C (2005). Inflammatory mediators are insufficient for full dendritic cell activation and promote expansion of CD4+ T cell populations lacking helper function.. Nat Immunol.

[40] Badovinac VP, Tvinnereim AR, Harty JT (2000). Regulation of antigen-specific CD8+ T cell homeostasis by perforin and interferon-gamma.. Science.

[41] Stepp SE, Dufourcq-Lagelouse R, Le Deist F, Bhawan S, Certain S (1999). Perforin gene defects in familial hemophagocytic lymphohistiocytosis.. Science.

[42] Feldmann J, Le Deist F, Ouachee-Chardin M, Certain S, Alexander S (2002). Functional consequences of perforin gene mutations in 22 patients with familial haemophagocytic lymphohistiocytosis.. Br J Haematol.

[43] Matloubian M, Suresh M, Glass A, Galvan M, Chow K (1999). A role for perforin in downregulating T-cell responses during chronic viral infection.. J Virol.

[44] Inaba K, Turley S, Yamaide F, Iyoda T, Mahnke K (1998). Efficient presentation of phagocytosed cellular fragments on the major histocompatibility complex class II products of dendritic cells.. J Exp Med.

[45] Kontgen F, Suss G, Stewart C, Steinmetz M, Bluethmann H (1993). Targeted disruption of the MHC class II Aa gene in C57BL/6 mice.. Int Immunol.

[46] Hogquist KA, Jameson SC, Heath WR, Howard JL, Bevan MJ (1994). T cell receptor antagonist peptides induce positive selection.. Cell.

[47] Barnden MJ, Allison J, Heath WR, Carbone FR (1998). Defective TCR expression in transgenic mice constructed using cDNA-based alpha- and beta-chain genes under the control of heterologous regulatory elements.. Immunol Cell Biol.

[48] Garrigan K, Moroni-Rawson P, McMurray C, Hermans I, Abernethy N (1996). Functional comparison of spleen dendritic cells and dendritic cells cultured in vitro from bone marrow precursors.. Blood.

[49] Ruedl C, Khameneh HJ, Karjalainen K (2008). Manipulation of immune system via immortal bone marrow stem cells.. Int Immunol.

[50] Robinson MJ, Ronchese F, Miller JH, La Flamme AC (2010). Paclitaxel inhibits killing by murine cytotoxic T lymphocytes in vivo but not in vitro.. Immunol Cell Biol.

[51] Bedoui S, Whitney PG, Waithman J, Eidsmo L, Wakim L (2009). Cross-presentation of viral and self antigens by skin-derived CD103+ dendritic cells.. Nat Immunol.

[52] Lee HK, Zamora M, Linehan MM, Iijima N, Gonzalez D (2009). Differential roles of migratory and resident DCs in T cell priming after mucosal or skin HSV-1 infection.. J Exp Med.

